# Effectiveness of CURA: Healthcare professionals’ moral resilience and moral competences

**DOI:** 10.1177/09697330231218344

**Published:** 2023-11-30

**Authors:** Malene van Schaik, H. Roeline RW Pasman, Guy AM Widdershoven, Janine De Snoo-Trimp, Suzanne Metselaar

**Affiliations:** Department of Ethics, Law and Humanities, 1209 Amsterdam UMC, Vrije Universiteit Amsterdam, Amsterdam, The Netherlands; Department of Public and Occupational Health, Amsterdam Public Health Research Institute, 1209 Amsterdam UMC, Vrije Universiteit Amsterdam, Amsterdam, The Netherlands; Department of Ethics, Law and Humanities, 1209 Amsterdam UMC, Vrije Universiteit Amsterdam, Amsterdam, The Netherlands

**Keywords:** Palliative care < topic areas, clinical ethics < topic areas, moral distress < topic areas, home care < areas of practice, care homes < areas of practice, quantitative research, moral competences, moral resilience

## Abstract

**Background:** Clinical ethics support instruments aim to support healthcare professionals in dealing with moral challenges in clinical practice. CURA is a relatively new instrument tailored to the wishes and needs of healthcare professionals in palliative care, especially nurses. It aims to foster their moral resilience and moral competences.

**Aim:** To investigate the effects of using CURA on healthcare professionals regarding their Moral Resilience and Moral Competences.

**Design:** Single group pre-/post-test design with two questionnaires.

**Methods:** Questionnaires used were the Rushton Moral Resilience Scale measuring Moral Resilience and the Euro-MCD, measuring Moral Competences. Respondents mainly consisted of nurses and nurse assistants who used CURA in daily practice. Forty-seven respondents contributed to both pre- and post-test with 18 months between both tests. Analysis was done using descriptive statistics and Wilcoxon signed rank tests. This study followed the SQUIRE checklist.

**Ethical considerations:** This study was approved by the Institutional Review Board of Amsterdam UMC. Informed consent was obtained from all respondents.

**Results:** The total Moral Resilience score and the scores of two subscales of the RMRS, that is, Responses to Moral Adversity and Relational Integrity, increased significantly. All subscales of the Euro-MCD increased significantly at posttest. Using CURA more often did not lead to significant higher scores on most (sub) scales.

**Conclusion:** This study indicates that CURA can be used to foster moral resilience and moral competences of healthcare professionals. CURA therefore is a promising instrument to support healthcare professionals in dealing with moral challenges in everyday practice.

## Introduction

Healthcare professionals (HCPs) frequently experience moral challenges in healthcare practice. Clinical Ethics Support (CES) aims to provide support or advice to HCPs in dealing with these challenges.^1,2,3^ CES has been related to improved quality of care,^4,5,6,7^ improved moral competences of HCPs,^5,8^ strengthening of moral resilience,^9^ and reducing moral distress.^10,11^ However, empirical evidence for these claims is limited.^6,12^ Providing evidence about the effectiveness of CES interventions is important to know whether (and which kind of) CES has effect on HCPs’ skills in dealing with these challenges and whether it impacts clinical practice,^7,13^ to provide reasons for organizations to implement CES,^6,8^ and to be able to compare different types of CES with each other.^14^

Two concepts have been specifically proposed as relevant outcomes of CES. One is moral competence. Moral competences are skills such as being aware of one’s own values, the ability to recognize moral conflicts, to communicate and reflect on these conflicts, to take the perspective and values of others into account, and the willingness and ability to act in accordance with this judgment in a morally responsible way and being accountable for one’s actions.^15^ Another relevant concept is “moral resilience,” that is, “the capacity of an individual to sustain or restore their integrity in response to moral adversity.”^16^ Moral resilience implies the capacity to navigate moral distress. Moral distress, that is, the psychological distress caused by a moral challenge,^17^ is frequently experienced by HCPs and poses a threat to the wellbeing of individual professionals as well as to quality of care.^18^

CURA is a relatively new CES instrument.^19^ It has been developed specifically for and with HCPs, in particular nurses who provide palliative care.^20^ It is designed to be used in a small group setting to reflect on morally challenging situations, although it can also be used individually (ibid.). In a feasibility and perceived effects study,^21^ nurses indicated that CURA helped them in building moral competences such as becoming aware of moral challenges, and reflecting on these challenges, as well as in dealing with moral distress. However, results were collected after nurses used CURA only once or twice. Medium- and long-term outcomes were not investigated. The current study aims to investigate the effects of CURA on professionals in palliative care after using it on a structural and frequent basis over a period of 18 months. Our main research question was: What is the association between the use of CURA and moral competences and moral resilience among HCPs?

## Methods

### Design, setting, and respondents

This study aims to investigate the effectiveness of CURA using a single group pre-posttest design with questionnaires following the SQUIRE checklist (supplementary file 1). It was combined with an implementation study in an effectiveness implementation design. In this hybrid design, effectiveness and implementation research are investigated together to test an implementation strategy while gathering data on the intervention’s impact on relevant outcomes.^22^ The results of the implementation study will be described elsewhere.

In line with the WHO, we conceive of palliative care as all treatment and care to promote the quality of life of patients with life-threatening illnesses or vulnerabilities. The provision of palliative care was an inclusion criterion for organizations to participate in this study. Most organizations also provided other types of care. In the Netherlands, where this study was based, all HCPs should be capable to provide palliative care as generalists. They are supported by specialists. Palliative care is delivered in various healthcare settings: hospitals, nursing homes, home care, and hospices. We included these four settings in our study. Participating organizations were: three hospitals, five nursing homes, three home care facilities, and two hospices. Between two to eleven HCPs from each organization participated in the study. They became acquainted with CURA as part of a training program for “CURA ambassadors.” This program aimed to enable participants to initiate, facilitate, and implement CURA within their own institution. The training consisted of an e-module, three interactive training sessions, practicing with organizing and facilitating CURA within their own organization, and writing an implementation plan. The core element of the program was using CURA either as participant or as facilitator. The respondents were selected by the organizations themselves. Inclusion criteria were affinity with clinical ethics, the intention to continue working for the organization for a longer period of time (>1 year), and the willingness to play an active role in implementation of CURA within their own organization.

### Intervention

CURA aims to strengthen moral resilience and moral competences of HCPs.^19^ CURA is a specific kind of moral case deliberation. Moral case deliberation (MCD) is an approach to clinical ethics support that seeks to foster methodically structured group dialogue among HCPs on a specific case that is experienced as morally troublesome.^23^ The experiences, moral intuitions, and judgments of participants are the main point of departure and reference in MCD, rather than abstract theories or concepts. MCDs are usually facilitated by an ethicist or a HCP who has received intensive training.^24^ CURA was developed as an accessible instrument to be used by HCPs themselves, either without or with a relatively short training.

CURA is an acronym for the four steps of the instrument: Concentrate; in which users describe the situation and their moral doubt(s). Unrush; in which users become aware of their first emotions, physical reactions, or judgments caused by the situation. Reflect; in which users venture into different perspectives by exploring what might be important for those involved (patient, family members, colleagues, themselves), and what is considered important in relevant guidelines and protocols. In the final step Act, users weigh and balance what has come up so far and judge what is most important to them, and what should be prioritized when taking action.^19^

### Data collection

The study used a questionnaire consisting of two validated scales. The first scale is the Rushton Moral Resilience Scale (RMRS).^25^ It measures the moral resilience of HCPs using 17 items on a four-point Likert scale. It consists of four subscales: Responses to Moral Adversity, Personal Integrity, Moral Efficacy, and Relational Integrity. A description of each subscale is provided in Table 1.

**Table 1.** Subscales of scales and reliability score.

(sub)scale	Description
RMRS Subscales^a^	
Responses to moral adversity	Assesses concepts such as powerlessness, being overwhelmed or feeling down by ethical adverse events
Personal integrity	Assesses the consistency with which a person is able to uphold their values in response to moral adversity
Moral efficacy	Assesses the extent to which a person feels capable to address ethical challenges and advocate for themselves and their values
Relational integrity	Assesses the interplay between personal and professional values, and the values of others
EURO-MCD Subscales^b^	
Individual moral competences	Assesses moral awareness, analytical skills and virtuous attitudes when dealing with moral doubts
Moral teamwork	Assesses the quality of dialogue and supportive relationships with other healthcare professionals
Moral action	Assesses concrete decisions and actual caring practices

^a^Heinze et al., 2021.

^b^De Snoo-Trimp et al., 2020a.

An overall score for Moral Resilience can be computed by taking the mean of all 17 items. Negatively worded items need to be coded reversed to ensure higher scores indicate higher moral resilience. The RMRS is validated and has an overall reliability of α=.83. It has been translated and culturally validated to fit the Dutch context.^26^

The second scale is the Euro-MCD 2.0.^27^ It measures the outcomes of Moral Case Deliberation (MCD) sessions in terms of what respondents experience in their daily work practice. The scale consists of three subscales: Individual Moral Competences, Moral Teamwork, and Moral Action, and entails 15 items in total. In this article, we use the term “Moral Competences” in a broad sense, including not only the first subscale but also the other two, because the items within Moral Teamwork and Moral Action also entail individual skills and hence, could be perceived as competences as well. The four-point Likert scale ranges from “strongly disagree” to “strongly agree.” The option “cannot take a stand” is also offered, in which case the score was set to “missing.” The scale is developed with input from diverse healthcare settings in European countries, among which the Netherlands. A Dutch version of the scale was therefore already available. A description of each subscale is provided in Table 1.

In addition to scoring on these two scales, respondents were asked to provide demographic details including age, gender, years of work experience, professional qualifications, work setting, and experience with using CURA.

**Figure 1.**
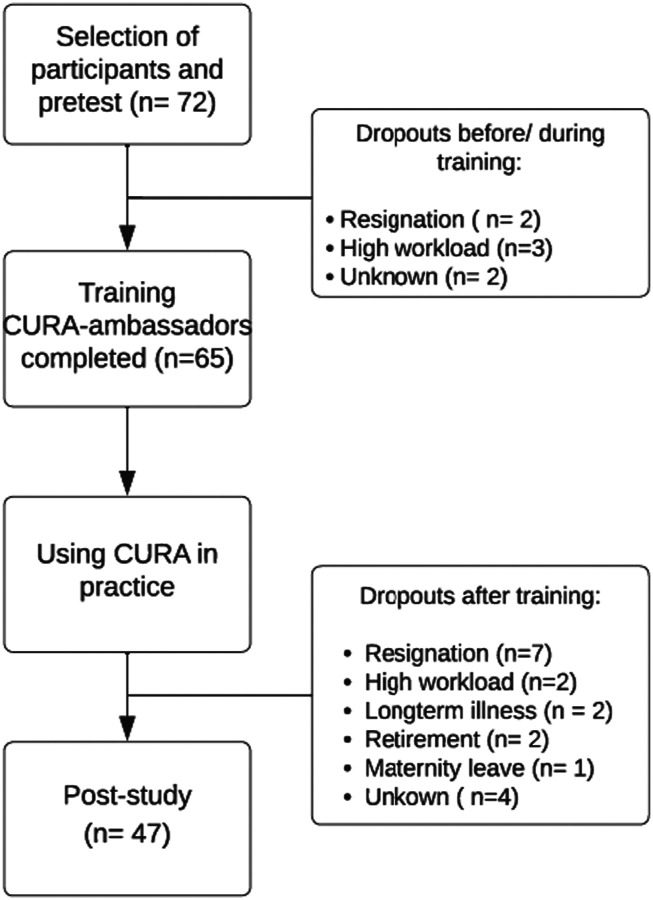
Respondent flow diagram.

Pre-test measurement was carried out between November 2020 and January 2021, before respondents started the training program. One organization (*n* = 5) started later due to COVID-19 restrictions and filled out the pretest survey in October 2021. Post-test measurement was planned 1.5 years after pre-test, to ensure respondents had adequate experience with CURA, and was carried out between April and August 2022. Respondents were contacted through email and reminders were sent out three times. Figure 1 shows the number of respondents and dropout scores. All respondents were assigned an anonymous code at pre-test, enabling us to pair outcomes of pre-test and post-test.

### Data analysis

Descriptive statistics were computed for all variables (frequencies, mean (SD)). Comparisons between the pretests and posttests were assessed using matched-pair Wilcoxon’s signed-rank tests. Data were analyzed using SPSS 26.0. Statistical significance was set at *p* < .05. Association analysis was done for healthcare setting, years of experience, and whether using CURA frequently lead to higher scores on the (sub) scales using descriptive statistics and Wilcoxon’s signed-rank tests. However, our sample size is too small to investigate statistical significant difference between settings.

We controlled for attrition bias by comparing the moral resilience and moral competences of the dropouts (*n* = 18) to the respondents that filled in both questionnaires (*n* = 47): no significant differences were found between these groups at baseline.

### Ethical considerations

The Institutional Review Board (IRB) of Amsterdam UMC was informed about the study and judged it not subject to the Medical Research Involving Human Subjects Acts, therefore it required no further ethical review by law (2020.478). Respondents of the study were informed in written documentation that they were participating in a scientific study. Written informed consent was provided by respondents at completion of the questionnaires.

## Results

### Participation rate and characteristics

Demographic and professional characteristics of respondents and dropouts are summarized in Table 2. Sixty-five respondents completed the training to become “CURA-ambassadors.” Of this group, 47 respondents completed both pre- and post-test questionnaires (response rate: 72%). Forty-two (89%) were female. Most respondents worked either in nursing home (*n* = 15, 32%) or hospital setting (*n* = 15, 32%). 34% had fewer than 5 years of work experience. Most respondents were Registered Nurse (*n* = 10, 21%), Nurse Assistant or Licensed Nurse Practitioner (*n* = 10, 21%), or Spiritual Counselors (*n* = 8, 17%). Eight (17%) of the nurses indicated to have specialized expertise in palliative care. The characteristics and pre-test results of the 18 dropouts were largely comparable to the 47 included respondents. However, most dropouts were younger than the pre-/post-intervention group and worked in a nursing home or hospital.

**Table 2.** Characteristics of respondents pre-post study.

Variable	Respondents (*n* = 47)	Dropouts (*n* = 18)
Age		
<30	3 (6%)	6/33%
31–40	6 (13%)	6/33%
41–50	11 (23%)	2/11%
51–60	17 (36%)	2/11%
>60	10 (21%)	1/6%
Years of work experience		^a^
0–5	16/34%	6/33%
6–10	7/15%	1/6%
11–20	16/34%	1/6%
>21	8/17%	2/11%
Female	42 (89%)	14/78%
Setting		
- Home care	10 (21%)	1/6%
- Nursing home	15 (32%)	9/50%
- Hospital	15 (32%)	8/44%
- Hospice	7 (15%)	0/0%
Profession		
- LPN and HC assistant	10/21%	6/33%
- RN	10/21%	6/33%
- Nurse with specialized expertise in palliative care	8/17%	1/6%
- Spiritual counselor	8/17%	0/0%
- Other^b^	11/22%	5/28%
Frequency usage of CURA		
• Every month or more	17/36%	N.A.
• Every 3 months	17/36%	
• Every 6 months	4/9%	
• Never	2/4%	
• Other	7/15%	
Number of times CURA has been used as a participant:		
• Not yet	4/9%	N.A.
• 1- 5 times	25/53%	
• 6- 10 times	11/23%	
• More than 10 times	7/15%	
As a facilitator:		
• Not yet	2/4%	
• 1- 5 times	18/38%	
• 6- 10 times	19/40%	
• More than 10 times	8/17%	

^a^missing: 8.

^b^Other professionals included physicians, a physician assistant, psychologists, social workers, a music therapist, team managers.

Thirty-six percent of respondents used CURA at least once every month, whereas another 36% used CURA about once in every 3 months. Thirteen percent used CURA once every 6 months or less. Furthermore, we asked respondents whether they used CURA as a participant or a facilitator. Respondents used CURA more often as a facilitator: 27 (57%) respondents used CURA more than six times as a facilitator, whereas 18 (38%) respondents used CURA more than six times as a participant.

### Rushton moral resilience scale

Results for all items and subscales are presented in Table 3. The mean score on the RMRS for Moral Resilience for the pre/post-intervention group (*n* = 47) was 2.9 pre-intervention and 3.1 post-intervention (*P *= <.001). Especially, scores on two subscales increased significantly pre-/post-intervention (*P *= <.001): Response to Moral Adversity and Relational Integrity. Two out of five items of the subscale Response to Moral Adversity increased significantly: *8. When a challenging ethical situation can’t be resolved, I find myself* “going through the motions” *in my job.(Reversed)* (*P *= <.001). And 4. *I am overwhelmed by persistent ethical conflicts. (R)* (*p* = .003). Relational Integrity consists of five items, of which three increased significantly: 11. *When faced with a difficult ethical challenge, I find myself doing or saying things that I later regret(R)* (*p =* .016); 13. *I would rather avoid conflict with those who have more authority than I do than act in accordance with my values* (R) *p =* .029) and 16. *My fear can cause me to act in a way that compromises my values* (R) (*P *= <.001).

**Table 3.** Results for (sub)scales and items pre-test and post-test (*n* = 47).

All items Noted as mean [SD]	Pre-test	Post-test	*p*-value
RMRS			
RMRS total	2.9 (.39)	3.1 (.37)	<.001
*Response to moral adversity*	2.7 (.50)	3.0 (.48)	<.001
2. Difficult ethical situations leave me feeling powerless (R)	2.4 [.87]	2.4 [.80]	.730
8. When a challenging ethical situation can't be resolved, I find myself “going through the motions” in my job (R)	2.9 [.86]	3.4 [.71]	<.001
4. I am overwhelmed by persistent ethical conflicts (R)	2.8 [.84]	3.3 [.72]	.003
5. After facing a challenging ethical situation, lingering distress weighs me down (R)	2.6 [.90]	2.8 [.85]	.154
14. When confronted with an ethical challenge, I push myself beyond what is healthy for me (R)	2.8 [.90]	3.0 [.83]	.109
Personal integrity	2.7 (.38)	2.8 (.40)	.253
17. No matter the situation I do what is consistent with my values	3.3 [.62]	3.2 [.66]	.076
1. In my professional role, my choices and behaviors consistently reflect my values	3.2 [.54]	3.2 [.62]	.819
6. I find it challenging to implement the decisions of others when it threatens my values (R)	2.0 [.81]	2.0 [.91]	.756
Moral efficacy	3.4 (.41)	3.5 (.39)	.129
7. When I am confronted by an ethical challenge, I am able to articulate the ethical conflict	3.4 [.65]	3.5 [.56]	.695
12. I am confident in my ability to reason through ethical challenges in my professional role	3.3 [.62]	3.5 [.62]	.077
9. I can think clearly when confronting an ethical challenge, even when I feel pressured	3.2 [.67]	3.2 [.75]	.455
3. I voice my ethical concerns in a way that others take seriously	3.6 [58]	3.8 [.41]	.012
Relational integrity	2.9 (.54)	3.2 (.54)	<.001
10. When others criticize my opinions, I compromise my values (R)	3.1 [.80]	3.3 [.74]	.175
11. When faced with a difficult ethical challenge, I find myself doing or saying things that I later regret (R)	2.6 [.77]	3.0 [.94]	.016
13. I would rather avoid conflict with those who have more authority than I do than act in accordance with my values (R)	3.1 [.83]	3.4 [.71]	.029
15. I tend to be distracted by others’ strong emotions when ethical conflicts occur (R)	2.6 [.97]	2.7 [.87]	.460
16. My fear can cause me to act in a way that compromises my values (R)	3.1 [.79]	3.5 [.75]	<.001
Euro-MCD			
Individual moral competences	3.4 (.33)	3.7 (.27)	<.001
1. I recognize a situation as being ethically difficult	3.4 [.52]	3.7 [.48]	.033
2. I am aware of other’s perspectives in ethically difficult situations	3.4 [.49]	3.7 [.48]	.027
3. I can identify the different values at stake in ethically difficult situations	3.4 [.56]	3.6 [.50]	.020
4. I can formulate arguments in favor of and against different courses of action in ethically difficult situations	3.3 [.55]	3.5 [.50]	.012
5. I listen with an open mind to others when discussing an ethically difficult situation	3.7 [.47]	3.9 [.31]	.008
6. I speak up in ethically difficult situations	3.5 [.59]	3.8 [.40]	<.001
Moral teamwork	3.3 (.73)	3.6 (.38)	.004
7. We openly express our viewpoints in ethically difficult situations	3.3 [.74]	3.5 [.55]	.060
8. We all have opportunities to express our viewpoint on ethically difficult situations	3.1 [.97]	3.6 [.54]	.004
9. We respect different viewpoints when discussing ethically difficult situations	3.5 [.72]	3.7 [.54]	.043
10. We feel secure to share emotions in ethically difficult situations	3.3 [.88]	3.5 [.59]	.062
11. We support each other when dealing with ethically difficult situations	3.5 [.59]	3.8 [.42]	.002
Moral action	3.3 (.50)	3.5 (.45)	.016
12. We make decisions on how to act in ethically difficult situations	3.0 [.66]	3.4 [.54]	.003
13. We base our decisions on moral considerations in ethically difficult situations	2.9 [.89]	3.3 [.56]	.003
14. We are responsive to the values and needs of patients and their families in ethically difficult situations	3.6 [.52]	3.6 [.57]	1.000
15. We are able to explain and justify our care towards patients and their families	3.5 [.59]	3.6 [.58]	.467

Differences between pre-test (*n* = 47) and post-test measurements were compared using Wilcoxon’s signed-rank tests variables are denoted as mean (SD). *Note: (R) these items were reverse coded: higher scores indicate higher moral resilience.

The scores on the subscales Personal Integrity and Moral Efficacy remained constant or increased slightly, but not significantly. One item of the RMRS decreased post-intervention: *17. No matter the situation I do what is consistent with my values* (Personal Integrity), although this was not significant either.

### Euro-MCD 2.0

The Euro-MCD 2.0 consists of three subscales: Individual Moral Competences, Moral Teamwork, and Moral Action. All these subscales increased significantly at post-test (*P*=<.050). Most scores on individual items increased significantly post-intervention. Items with the highest increase were: *6. I speak up in ethically difficult situations (P*=<.001, Individual Moral Competences) *12. We make decisions on how to act in ethically difficult situations* (*p* = .003, Moral Action), and *13. We base our decisions on moral considerations in ethically difficult situations* (*p* = .003, Moral Action)*.* However, four items did not increase significantly, among which *14. We are responsive to the values and needs of patients and their families in ethically difficult situations* (Moral Action) and *15. We are able to explain and justify our care towards patients and their families* (Moral Action). The option “Cannot take a stand” was given 7 times maximum at pre-test, and a maximum of 3 at post-test.

### Setting, years of experience, and frequency in use

Specified to setting, the total moral resilience score increased in all healthcare settings, although not significantly. The smallest increase occurred in the hospital setting (increase of 0.1), and the greatest increase was observed in both home care and hospice settings (0.3). Sixteen respondents were beginner HCPs with fewer than 5 years’ experience. Their median scores on most subscales were similar to HCPs with more experience, both on pre-test and post-test.

Furthermore, as Table 4 shows, using CURA more frequently (>5 times) did not result in significantly higher scores on subscales. Using CURA either as a participant or a facilitator shows a similar pattern in scores on subscales.

**Table 4.** Years of work experience, frequency of using CURA as participant and as facilitator.

	Moral resilience pretest	Moral resilience posttest	Individual moral competences pretest	Individual moral competences posttest	Moral teamwork pretest	Moral teamwork posttest	Moral action pretest	Moral action posttest
Years of experience								
5 years or less (*n* = 16)	3.0 (.37)	3.2 (.29)	3.6 (.36)	3.7 (.20)	3.2 (.92)	3.5 (.40)	3.3 (.59)	3.4 (.47)
More than 5 (*n* = 31)	2.9 (.41)	3.1 (.40)	3.4 (.30)	3.7 (.30)	3.3 (.63)	3.7 (.36)	3.3 (.46)	3.5 (.44)
Using CURA as participant								
5 times or less (*n* = 29)	3.0 (.39)	3.2 (.39)	3.5 (.34)	3.7 (.26)	3.2 (.83)	3.7 (.38)	3.3 (.59)	3.5 (.49)
More than 5 (*n* = 18)	2.9 (.40)	3.0 (.30)	3.4 (.32)	3.7 (.28)	3.4 (.54)	3.6 (.38)	3.3 (.31)	3.5 (.55)
Using CURA as facilitator								
5 times or less (*n* = 20)	2.9 (.33)	3.1 (.30)	3.4 (.29)	3.6 (.26)	3.3 (.70)	3.6 (.43)	3.2 (.54)	3.5 (.44)
More than 5 (*n* = 27)	2.9 (.44)	3.2 (.41)	3.5 (.36)	3.7 (.26)	3.3 (.77)	3.6 (.34)	3.3 (.48)	3.5 (.47)

Median [interquartile range] * differences between pre-test and post-test were compared using Wilcoxon’s signed-rank tests.

## Discussion

The aim of this study was to investigate whether and how a specific CES instrument, CURA, impacts HCPs’ moral resilience, and moral competences. This study demonstrates that respondents using CURA over a 1.5 year time period score significantly higher on Moral Resilience and Moral Competences, compared to before using CURA.

### Improved moral resilience

The total score of the RMRS scale and two out of four subscales improved significantly. On that basis, we can conclude that the moral resilience of participants increased. These subscales are Relational Integrity and Response to Moral Adversity. The former subscale measures the interplay between personal values and the values of others, the latter concepts such as powerlessness, being overwhelmed or feeling down by moral challenges. The improvement on the subscale Relational Integrity may be related to a specific aim of CURA to encourage dialogue among respondents, as well as openness to and venturing into the perspectives of others.^19^ The significant improvement on the subscale Responses to Moral Adversity may be related to the focus on paying attention to emotions and initial responses to ethical challenges, which is an essential element of CURA.

Another study^28^ using the RMRS found significant associations between specific subscales of the RMRS and subscales of MBI-HSS,^29^ a widely used survey to measure burnout, whereas other subscales did not show a significant relationship. For instance, higher Relational Integrity was related to lower scores on the subscale Emotional Exhaustion, but not to other domains of burnout. They suggest that the subscales of the RMRS represent unique constructs and researchers must consider which subscale is most relevant to their question of interest. Furthermore, they conclude that factors of moral resilience can be strengthened by providing tailored interventions to foster moral resilience of HCPs. This will help to create healthy and sustainable workforce in healthcare.

The scores on the other two subscales did not improve significantly in our study. These are Personal Integrity, measuring the consistency with which a person is able to uphold their values when confronted with an ethical challenge, and Moral Efficacy, measuring the extent to which one feels confident and capable to address ethical challenges and advocate for their own values. A possible explanation is that CURA is not so much aimed at upholding one’s values or defending them, regardless of the situation. Rather, CURA invites participants to reflect on one’s values while taking into consideration the values of others. This may lead to a reconsideration of what is important in the situation, and possibly to prioritizing different values. This may explain that the outcomes of these two subscales did not change.

### Improved moral competences

All subscale scores on the Euro-MCD 2.0 increased significantly. All scores comprising the subscale Individual Moral Competences increased significantly, with high mean scores ranging up to 3.9 on a four-point scale. The high scores on pre-test as well as post-test could partly be explained by respondents overrating themselves, which is a common pitfall in self-assessment.^30^ Items on the Euro-MCD 2.0, specifically the items measuring Individual Moral Competences might be prone to overrating, as they measure the self-assessments of specific skills and are formulated affirmatively, for instance item *5: I listen with an open mind to others when discussing an ethically difficult situation*, However, while self-reporting holds limitations, it is also important to investigate individuals’ views on their moral competences, as these are difficult to capture through objective measurements like patient-reported quality of care scores.^31,32^

At post-test, respondents experienced more opportunities to share their viewpoints on ethically difficult situations, resulting in higher scores on Moral Teamwork. This is in line with the aim of using and implementing CURA in an organization, that is, to establish opportunities and spaces for dialogues. Multiple studies have highlighted the importance of frequent debriefing sessions and a safe ethical work environment.^33,34,35^ This can reduce intent to leave and increase job satisfaction.^34,36^ Furthermore, an increase in opportunities to discuss ethically difficult situations (i.e., by Moral Case Deliberation) is found to lead to better collaboration and higher moral reflexivity in daily practice as well.^31^

Most scores on the Euro-MCD improved significantly. However, two items measuring Moral Action remained constant. These were: 14. *We are responsive to the values and needs of patients and their families in ethically difficult situations,* and: 15. *We are able to explain and justify our care towards patients and their families.* While the scores on pre-test were already high (3.6 and 3.5), the results show no improvement on these items. Possibly, respondents use CURA primarily for their own support during ethically difficult situations, and do not relate CURA directly to communication with patients and family members. This aim of CURA, that is, that CURA may help improve patient care in morally difficult situations, might need more attention in the presentation of the instrument, for instance during training.

### Small variations in outcomes between groups

In all settings, Moral Resilience and all subscales of the Euro-MCD increased, though not significantly. Statistical analysis of differences between settings was not possible due to small frequencies. The increase in mean total scores of Moral Resilience were highest for home care and hospice setting (0.3). This could be explained by the fact that these settings generally work in smaller teams and have a less hierarchical organization than hospitals and nursing homes, which might make it easier to take initiatives to use CURA and to discuss their moral doubts more openly in smaller groups.

There was no significant difference between the scores of HCPs with less or more work experience: their scores followed a similar trend. While some studies have found higher moral distress among HCPs with more years of experience,^37,38^ other studies have found that unexperienced HCPs are at greater risk of stress, burnout, and higher intention to leave the profession^39,40,41^ and are therefore more in need of support.^41^ In our study, the selection of respondents could explain why we found no difference between beginner and more experienced HCPs. The respondents had affinity with ethics (support). Therefore, the respondents of this study are not representative for the entire population of HCPs. Novice HCPs that were enrolled could have had higher moral resilience and moral competences than the general population of novice HCPs, due to their affinity with ethics support from early on in their career. Furthermore, it could also be explained by attrition bias: the attrition rate among novice HCPs was much higher than HCPs with more years of experience. This could have led to an inaccurate representation of the novice group compared to the more experienced HCPs.

We did not find subgroup differences for respondents that had used CURA more than five times, either as a facilitator or participant. This finding may be explained by the “ceiling effect.” Scores were already high on baseline for all participants. However, still some effect was measurable after using CURA for a few times, after which the “ceiling” was reached, making discrimination for subjects using CURA more frequently impossible. This finding is in line with another study using the Euro-MCD,^31^ which found no statistical difference on items when using the Dilemma Method (another MCD instrument) more frequently.

### Implications for clinical practice

HCPs in palliative care experience high levels of moral distress, which contributes to high levels of burnout, high turnover rates and absenteeism and ultimately to lower quality of care.^25,42^ CES, and more specifically MCD, is proposed as a means to minimize moral distress and its negative consequences.^43,44^ This current study further adds to the empirical evidence on the outcomes of CES. Our results indicate that CURA is associated with improvement of moral resilience and moral competences. Other studies have also found CES to improve HCPs’ Moral Competences and Moral Resilience,^9,31,34,44,45^ for instance, by strengthening their analytical skills, and by creating better collaboration between co-workers.

Yet, it remains unclear whether CES also leads to better quality of care. Empirical evidence for concrete effect of CES on quality of care remains limited and is mostly based on qualitative studies or single cases.^34,46,47^ Quality of care is hard to measure and difficult to improve.^48^ Specifically within the context of CES, the concept of “quality of care” or “good care” is viewed as context-dependent.^49^ (Re)defining what constitutes as quality of care in a specific case is often a central question during a MCD session. Therefore, it is challenging to measure the impact of MCD on quality of care with standardized questionnaires.^49^ However, some authors have proposed a strategy to reach quality improvements in patient care through CES by integrating regular CES sessions in clinical routines.^6,50^ CURA, being low threshold and relatively easy to apply in daily practice, seems a promising instrument to have regular group sessions to reflect on moral doubts. The challenge remains, however, to measure the effect on patient care.

## Strengths and limitations

A strength of this study is the inclusion of several (palliative) care settings. CURA is intended to be used in a wide range of palliative care settings. While our data set was too small to make subgroup comparisons, we found that CURA had effect in all included care settings. A second strength of this study is the relatively long period between pre- and post-test. This limits the risk of a “testing-effect,” that is, when scores on the post-test are higher due to exposure to the pre-test.^51^

This study also holds some limitations. First, we did not use a random sample of respondents. Therefore, our sample is not representative for the total population of HCPs. Our respondents already had affinity with CES and therefore possibly had higher scores on Moral Resilience and Moral Competences at baseline. However, a study in the U.S. which included a wider variety of HCPs (*n* = 702) from multiple U.S. hospitals revealed high baseline scores on Moral Resilience as well.^25^ Mean scores on subscales ranged from 2.7 to 3.7 on the RMRS subscales, which is similar to our findings at baseline. Moreover, even in our biased population, the results increased at post-test. This might indicate that our results are an underestimation and the actual results in a general population of HCPs may even be higher.

Second, respondents received training on how to use CURA. The core element of the training program is to practice with CURA as a participant and a facilitator, which will have influenced our outcome measures. Therefore, the question remains whether using CURA has similar effects on HCPs who did not attend the training.

Third, it is difficult to be certain that positive changes can be attributed to the use of CURA. First of all, we did not use a control group. Furthermore, whereas other respondents used CURA more frequently, about 60% of our respondents reportedly used CURA as a participant between 1 and 5 times, and about 40% of them used it as facilitator between 1 and 5 times during the 1.5 years study timeframe. Although it is not extraordinary to use a CES instrument on an incidental rather than on a day-to-day basis, these frequencies complicate establishing whether it is indeed CURA that impacted positive outcomes on moral resilience and moral competences. Other factors could be of influence as well. It could also be the case that the benefits of CURA go well beyond the formal CURA-sessions. We know from some CURA-ambassadors that they also started to use CURA very informally, or incorporate steps of the CURA method in the way they deal with moral challenges on a more intuitive, day-to-day basis. Qualitative research on the effects of CURA could provide more insight in how and whether this has impacted them.

Our results might be influenced by the COVID-19 pandemic. Our pre-test was conducted during the second COVID-19 wave (November 2020–January 2021), and the post-test was conducted in the aftermath of the pandemic (April–August 2022). To date, there is no consensus on how feelings of moral distress and resilience of HCPs developed during the pandemic. Some studies indicate HCPs were less resilient at the end of the pandemic than during the second wave,^52,53^ due to the emotional burden and understaffing that became increasingly problematic. However, other studies found that most HCPs became “stress resistant” or remained resilient during the pandemic.^54,55^ Hence, it remains unclear whether respondents were most affected by moral distress related to COVID-19 at pre-test or post-test, as studies on the long-term effects of COVID-19 on moral distress and mental health of HCPs are still emerging. Future research should corroborate our results. This requires conducting a larger study with a larger sample size and preferably working with a control group, allowing for subgroup analysis of different care settings and different professional groups.

Finally, this study focused on a quantitative approach. While our findings are promising, our insights into the effectiveness of CURA could benefit from qualitative research as well. Adding qualitative data may contribute to gaining an in-depth understanding of participants experiences of *how* CURA can be effective in strengthening moral competences and moral resilience. Therefore, this will be a focus of future research on the effectiveness of CURA.

## Conclusion

The aim of this study was to investigate the effects of using CURA, a CES instrument, on moral resilience and moral competences of HCPs. The results are promising. We found statistically significant changes after using CURA for 18 months on the total Moral Resilience score and on the two subscales Responses to Moral Adversity and Relational Integrity. All subscales of the Euro-MCD 2.0 (Individual Moral Competency, Moral Teamwork, and Moral Action) increased as well. This study indicates that CURA can be used to strengthen both the moral resilience and moral competences of HCPs. It adds to our knowledge and strategies to foster a resilient workforce in healthcare, which is highly relevant given present-day challenges such as personnel shortages, high absenteeism, high work pressure, and concerns about the feasibility of our health care system. Fostering morally competent HCPs, who can successfully navigate moral complexities and deal with moral challenges in everyday practice, may also foster quality of care for patients and family members. Future research on CURA should follow up on this study with a larger sample size and with a control group.

## Supplemental Material

Supplemental Material - Effectiveness of CURA: Healthcare professionals’ moral resilience and moral competences Supplemental Material for Effectiveness of CURA: Healthcare professionals’ moral resilience and moral competences by Malene van Schaik, H. Roeline RW Pasman, Guy AM Widdershoven, Janine De Snoo-Trimp, and Suzanne Metselaar in Nursing Ethics
